# Assessing the Impact of Polyamide Nanofibrous Material Areal Weight on *Lacticaseibacillus rhamnosus* Biofilm Formation and Resistance to Storage Conditions and Contamination

**DOI:** 10.1021/acsomega.5c01042

**Published:** 2025-10-07

**Authors:** Vaclav Peroutka, Marta Stindlova, Vera Jencova, Vera Lacinova, Jana Jiresova, Katerina Demnerova, Simona Lencova

**Affiliations:** † Department of Biochemistry and Microbiology, 52735 University of Chemistry and Technology, Prague 166 28, Czech Republic; ‡ Department of Chemistry, Faculty of Science, Humanities and Education, 48261 Technical University of Liberec, Liberec 461 17, Czech Republic; § Department of Physics and Measurements, University of Chemistry and Technology, Prague 166 28, Czech Republic

## Abstract

Probiotic biofilms are considered the fourth most advanced generation of probiotics. To maximize the benefits of probiotic biofilms, suitable carriers ensuring bacterial viability during storage are being sought. The use of nanofibrous platforms is beginning to appear as one of the most promising approaches. We investigated the influence of three polyamide (PA) nanofibrous materials with different areal weights (5, 11, 27 g/m^2^) and the resulting morphological properties on the biofilm formation of *Lacticaseibacillus rhamnosus* ATCC 9595 and its tolerance to various conditions. PA promoted biofilm formation more than the reference material, polystyrene. PA’s areal weight influenced the biofilm biomass amount, phenotype, and structure; PAs with a high areal weight promoted biofilm formation. Further, we examined the tolerance of matured biofilms on the PAs to various external conditions: (i) storage temperature (−20, 4, 21 °C), environment (aqueous/dry), and time (0–35 days), (ii) pH (2, 4, 6, 7, 8 and 10), and (iii) bacterial contamination by *Staphylococcus aureus* and *Escherichia coli*. Generally, PAs increased biofilm resistance, and the areal weight of the PA played a crucial role in it. The PA with the highest areal weight (27 g/m^2^) provided the highest long-term stability and tolerance of the biofilm and thus was confirmed to be the most suitable tested nanomaterial. The overall results suggest that the presented PAs could be suitable carriers of probiotic biofilm, enabling large-scale production. We also highlight the need for further research on the influence of nanomaterials’ morphology on microbial interactions, possibly enabling target modification for a particular use.

## Graphical abstract

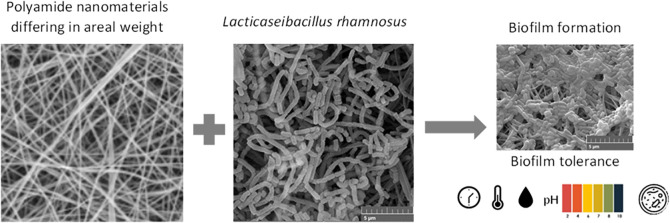

## 1 Introduction

Probiotics are defined as “live microorganisms which, when administered in adequate amounts, confer health benefits on the host”.^1^ Generally, the beneficial effects on the host are based on (i) the induction of immunomodulation, (ii) the protection against physiological stress, (iii) the suppression of pathogens, (iv) microbiome modulation, and (v) improvement of the barrier function of the gut epithelium.^2^ The ability of probiotics to shape the host′s immune defense, restore microbial balance in the intestines, and detoxify microbial toxins has led to their widespread use in the prevention and treatment of infectious diseases and chronic inflammation of the digestive tract.^3^

The consumption of probiotics to promote health and well-being continues to rise, making them one of the most widely consumed food supplements worldwide.^2^ With the growing probiotic industry, the demand for both quantity and quality, specifically in terms of viability and resistance of probiotic bacteria, is also increasing. Commercialized probiotic products are typically made from planktonic cells, which represent one of the two essential forms of bacterial life, along with biofilms. The use of planktonic cells is attributed to conventional manufacturing methods. However, the large-scale production of planktonic probiotic cells remains a significant challenge in industrial settings. The probiotic properties of these cells are easily influenced not only by growth and storage conditions^4^ but also, more importantly, by cell viability, which is a fundamental prerequisite for their probiotic functionality.^5^

Given these limitations, the use of biofilms, the second form of probiotic life, has gained increasing attention from the scientific community. Biofilms, described as gatherings of microbial cells irreversibly associated with a surface, are more resistant to environmental factors than planktonic cells and are, therefore, the predominant form of microbial life.^6^ In the life cycle of probiotics, biofilms are the naturally preferred life form, as adhesion and colonization are essential for the immunoregulatory function of probiotic microorganisms.^7^ Probiotic biofilms have been intensively studied over the past decade, with numerous studies confirming the advantages of biofilm-forming cells over planktonic ones. For instance, biofilm cells of *Lacticaseibacillus casei*,*Lactiplantibacillus plantarum*, and *Limosilactobacillus fermentum* have shown enhanced anti-inflammatory effects compared to their planktonic forms.^7,8^ Biofilms of *Limosilactobacillus reuteri* have been found to reduce the severity of *Clostridium difficile* infection,^9^ and *Lacticaseibacillus paracasei* biofilms have been shown to enhance host health.^10^ Based on these findings, probiotic biofilms are currently considered the fourth most advanced generation of probiotics.^11^

Despite the benefits of probiotic biofilms, their large-scale production, providing high cell viability, remains an urgent need to be addressed.^11^ Electrospun nanofibrous materials are currently considered for use in various biomedical applications and as one of the most promising carriers for probiotic biofilms due to their unique properties such as high surface area, porosity, and the ability to modify their structure as needed.^12,13^ Additionally, these materials can be produced at scale in a relatively simple and cost-effective manner.^6,11^

However, only a few studies have focused on the interactions between nanofibrous materials electrospun from various polymers and probiotic biofilms. For example, cellulose acetate nanofibrous membranes have been shown to be effective scaffolds for *L. plantarum* biofilm formation, exhibiting high gastrointestinal resistance.^14^ In another recent study, ethyl cellulose nanofibers were tested as scaffolds for the *L. plantarum* biofilm, with biofilms demonstrating improved gastrointestinal and thermal tolerance compared to planktonic cells.^15^ Next, cellulose acetate nanofibrous membranes were found to support *L. paracasei* biofilms, offering long-term survival (up to 35 days) and high tolerance to extreme pH conditions and antibiotics, with the morphology of the nanomaterials influencing biofilm formation.^11^ These studies highlight the potential of nanofibrous materials as probiotic biofilm carriers as well as the considerable research gap due to the novelty of this approach. So far, only a limited number of nanomaterials and probiotic bacteria have been tested, and little attention has been paid to the targeted modulation of biofilm formation through the modification of the nanomaterial morphology.

Polyamide (PA) nanofibrous materials possess specific properties that could be exploited in the production of carriers for probiotic bacteria. In particular, they are outstanding for their mechanical resistance, which makes them reusable. Furthermore, they are biocompatible, which allows their wide application in medicine.^16^ The homogeneity of the softened PA nanomaterials is also an important property in terms of the production of well-defined materials, compared to the widely used polyester nanomaterials, where homogeneity is difficult to achieve.^17,18^ PA materials are thus also suitable model materials for studying the effect of specific morphology, such as fiber diameter or areal weight, on cells.

Given the need for a deeper understanding of the interactions between nanofibrous materials and probiotic biofilms, we present the first study to use PA nanofibrous materials as carriers for the biofilm of *Lacticaseibacillus rhamnosus*, one of the most widely used lactic acid bacteria with probiotic potential.^19^ The scope of this study includes the investigation of the influence of PA nanomaterial areal weight on biofilm formation, the evaluation of biofilm tolerance under various storage conditions, pH, and bacterial contamination, and the statistical analysis of the data to provide recommendations for future research.

## 2 Materials and Methods

### 2.1 Nanomaterial Preparation and Characterization

A set of three PA nanomaterials differing in areal weight was prepared and characterized as described in our previous study^6^ (Table 1). Briefly, the PA nanomaterials were prepared from 15% (w/w) PA6 (Ultramid B 27 E 01, BASF) polymer solution in a solvent system of acetic acid and formic acid (Penta, Czech Republic) in the ratio of 2:1 (v/v) by the needleless electrospinning method using a Nanospider NS 1 S500 U device (Elmarco, Liberec, Czech Republic). The areal weight was controlled by the withdrawal speed (areal weight is increased due to decreasing withdrawal speed: PA5 0.355m/min; PA11 0.22m/min, PA27 0.13 m/min), thus determining the density of fibers in the nanomaterial. The prepared PA nanomaterials were characterized by scanning electron microscopy (SEM) using Tescan Vega3 SB Easy Probe (TESCAN, Czech Republic) and NIS Elements software (Nikon, Japan). The thickness of the PAs was determined using XX Corp ID-C112XB (Mitutoyo, Japan).

**Table 1:** PA Nanomaterials Used in This Study and Their Characteristics^a^

**sample**	**material**	**areal weight** **[g/m^2^]**	**fiber diameter [nm]**	**air permeability** **[l/m^2^/s]**
**PA 15% 5** g/m^**2**^ **(PA5)**	PA 15%	5.25 ± 0.38	236.2 ± 66	16.0 ± 11.7
**PA 15% 11** g/m^**2**^ **(PA11)**	PA 15%	11.27 ± 0.71	236.2 ± 66	9.9 ± 3.1
**PA 15% 27** g/m^**2**^ **(PA27)**	PA 15%	26.82 ± 0.78	236.2 ± 66	5.8 ± 2.1

a The presented PA materials were characterized within our previous study^6^ from which the characteristics (areal weight, fiber diameter, and air permeability) were taken.

### 2.2 Bacterial Cultivation

The bacterial cultures *L. rhamnosus* ATCC 9595 (eq CCM 1828), *Staphylococcus aureus* ATCC 25923 (eq CCM 3953), and *Escherichia coli* ATCC 25922 (eq CCM 3954), representing standard reference strains, were obtained from the Czech Collection of Microorganisms (CCM, Czech Republic). The pure culture of *L. rhamnosus* was grown in the De Man–Rogosa–Sharpe broth (MRS) and MRS agar (Merck, Germany) at 37 °C for 48 h and used in biofilm studies. Pure cultures of *S. aureus* and *E. coli* were grown in tryptone soy broth (TSB) and on TS agar (Merck, Germany) at 37 °C for 24 h and used in the tolerance assay.

### 2.3 Biofilm Formation

Sterile (low-temperature ethylene oxide, laboratory temperature, 12 h cycle) PA nanomaterials of size 1 cm × 1 cm were used as biofilm carriers. A piece of PA was placed in a sterile 12-well microtiter plate (Greiner Bio-One, Austria) containing 3 mL of bacterial suspension in MRS with an optical density of 0.5 McF (McFarland; McF stands as a reference turbidimetric unit of optical density and corresponds to the concentration of bacterial cells in medium). The samples were cultivated at 37 °C for 48 or 72 h under aerobic conditions. Control biofilms were formed in the same way on polystyrene (PS), the material of the microtiter plate.

### 2.4 Determination of Biofilm-Forming Cells

Since the quantification of the total biofilm biomass would be very difficult to measure in the case of cultivation on laboratory-scale materials, or burdened with considerable error, the biofilm quantification was performed, neglecting the mass of the extracellular matrix, by calculating the number of viable cells, that is, colony-forming units, CFUs. CFU enumeration was selected as a suitable approach for the quantification of biofilm-forming cells according to our previous study.^6^ PA nanomaterials with formed biofilms were washed five times with a sterile saline solution (0.9% solution of NaCl in distilled water) and dried for 45 min at laboratory temperature. Then, 1 mL of a sterile saline solution was added to all wells. The plates were sonicated for 3 min to release the biofilm-forming cells. The obtained suspension was decimally diluted up to the ninth dilution, and droplets of 20 μL of individual dilutions were deposited on the surface of MRS agar in accordance with the Miles and Misra method of CFU enumeration. After cultivation (37 °C, 48 h, aerobic conditions), CFU/cm^2^ and the percentage of grown biofilm relative to the reference materials, PS, were evaluated.^6^

### 2.5 Tolerance Assay

To test the resistance of biofilms formed on PA, a tolerance assay comprising the following partial analyses was designed. In all the partial analyses, PA with matured biofilms (48 h cultivation) prepared according to the description given in Section 2.3 were used. PS with matured biofilms were used as controls.

First, the influence of storage conditions on the viability of biofilm-forming cells was verified. The biofilms formed on the PA nanomaterials and PS, as described in Section 2.3, were rinsed three times with a sterile saline solution and put into sterile 12-well plates. The long-term viability (stored 0–35 days) of the biofilm-forming cells was tested under the following conditions: (i) various temperatures of −20, 4, and 21 °C and (ii) various environments of aqueous or dry. The PAs stored in the aqueous environment were stored in 2 mL of sterile saline solution. In total, six storage condition comninations for each type of the PA and PS as a reference material were tested: (i) PA in a saline solution stored at −20 °C, (ii) PA in a dry plate stored at −20 °C, (iii) PA in a saline solution stored at 4 °C, (iv) PA in a dry plate stored at 4 °C, (v) PA in a saline solution stored at 21 °C, and (vi) PA in a dry plate stored at 21 °C. At regular intervals, every 7 days, the number of viable biofilm-forming cells is expressed as CFU/cm^2^, and the survival rate was determined as described in Section 2.4.

Next, we evaluated biofilm tolerance to acidic and alkaline pH levels according to published protocols.^11^ Briefly, biofilms formed on the PAs and PS, as described in Section 2.3, were rinsed three times with a sterile saline solution. The rinsed PA nanomaterials were put into 2 mL of sterile solutions of pH 2, 4, 6, 7, 8, and pH 10 prepared by the addition of small amounts of HCl or NaOH to sterile saline solution, separately. After the incubation (10 min, laboratory temperature), the PAs were rinsed with sterile saline solution and sonicated for 3 min in 1 mL of sterile saline solution to release biofilm-forming cells from nanomaterials. The quantification of viable CFUs was performed as described in Section 2.4, and the survival rate was determined as described in Section 2.4.

Finally, we determined the biofilm tolerance to external bacterial contamination. Biofilms formed on the PA nanomaterials and PS, as described in Section 2.3, were rinsed three times with a sterile saline solution and put into a sterile 12-well plate. Suspensions of *S. aureus* and *E. coli* (1 mL, optical density 0.5 McF) were separately added to the PA nanomaterials and PS with a probiotic biofilm. After cultivation (37 °C, 24 h), the PAs and PS were rinsed with a sterile saline solution and sonicated for 3 min in 1 mL of sterile saline solution to release the biofilm-forming cells from nanomaterials. The quantification of viable CFUs was performed as described in Section 2.4. To distinguish and quantify the bacteria present in the samples, serial dilutions were plated on selective media. De Man–Rogosa–Sharpe agar (MRS; Merck, Germany) was used for lactobacilli quantification, Baird-Parker agar (BP; Merck, Germany) for *S. aureus*, and Tryptone Bile X-glucuronide agar (TBX; Merck, Germany) for *E. coli*. The percentage representation of *L. rhamnosus* and a particular pathogen was evaluated.

### 2.6 Scanning Electron Microscopy

PA nanomaterials with matured biofilms (as described in Section 2.3) and treated as described in Section 2.5 were analyzed by SEM.^20^ Briefly, the PA samples were rinsed with phosphate-buffered saline (PBS), fixed in frozen ethanol (99.85%, Penta, Czechia) at 4 °C for 15 min, and dewatered with ethanol series with gradually increasing ethanol concentration (60–99.8%); the PA samples were left for 5 min in each ethanol concentration. After drying at laboratory temperature for at least 24 h, the samples were coated with gold (10 nm) and observed using a SEM Tescan Mira 3 LMH scanning electron microscope (Tescan, Czechia) equipped with a Schottky cathode at 15 kV accelerating voltage.

### 2.7 Statistical Data Analysis

All of the experiments were performed in at least nine replicates. Data analysis was performed in the R programming language in the RStudio program. The data sets obtained were subjected to Dean-Dixon’s Q-test for discarding outliers and the Shapiro–Wilk test for the verification of the normal distribution of data. The data were considered normally distributed at *p* > 0.05. For data analysis and comparison, an unpaired two-sample *t* test and multiple comparisons by one-way analysis of variance (ANOVA) were used at a significance level α = 0.05 and 0.01. If a significant result was found, Tukey Honest Significant Differences (Tukey HSD) was calculated to perform multiple pairwise comparisons of groups’ means. Data analysis was applied to test the following hypotheses (H1–H4):

**H1**: PA nanomaterials promote the biofilm formation of *L. rhamnosus* more than PS.

**H2**: Areal weight of PA nanomaterials influences the biofilm formation of *L. rhamnosus*.

**H3**: *L. rhamnosus* cells in the biofilms formed on PA remain viable longer during storage than the cells in biofilms formed on PS.

**H4**: Tolerance of *L. rhamnosus* biofilms formed on PA to external influences is impacted by areal weight.

## 3 Results and Discussion

### 3.1 Influence of PA Areal Weight on Biofilm Formation

PA nanomaterials were selected as the appropriate platforms for *L. rhamnosus* biofilm formation due to their high mechanical resistance, biocompatibility, nonbiodegradability, and stability.^16^ As previous research has confirmed the significant role of nanofibrous materials morphology in microbial interactions,^20–23^ we tested a set of three PA nanomaterials differing in areal weight, determining the density of fibers in the nanomaterial and the final thickness (PA5 0.024 mm, PA11 0.52 mm, PA27 0.148 mm).

The areal weight was controlled by the withdrawal speed during electrospinning; therefore, the resulting materials differ only in areal weight (5, 11, and 27 g/m^2^). The resulting PA nanomaterials exhibited homogeneous structures with a fiber diameter of 236 nm (with a narrow distribution of ± 66 nm) and the absence of defects.^6^ Further, the hypotheses **H1** “PA nanomaterials promote biofilm formation of *L. rhamnosus* more than PS” and **H2** “Areal weight of PA nanomaterials influences biofilm formation of *L. rhamnosus*” were tested.

*L. rhamnosus* formed a biofilm on all of the tested materials (Figure 1). No difference in the amount of biofilm formed at 48 and 72 h was observed (*p* < 0.05). In both cases, biofilms formed on PAs contained between 1.4 × 10^7^ ± 3.2 × 10^6^ and 1.5 × 10^8^ ± 4.3 × 10^7^ CFU/cm^2^ during 48 h cultivation and between 1.8 × 10^7^ ± 5.9 × 10^6^ and 1.2 × 10^8^ ± 3.4 × 10^7^ CFU/cm^2^ during 72 h cultivation. PA nanomaterials promoted biofilm formation more than the reference material, PS (*p* < 0.05). The observed variability of quantitative biological testing reflects the inherent nature of experiments based on biological systems, which are inherently chaotic to a large extent. Biofilms, in particular, as complex microbial communities, exhibit spatial heterogeneity, microniches, and stochastic population dynamics. CFU counts obtained from such populations are subject to stochastic variation. Based on the results, the hypothesis for **H1** “PA nanomaterials promote biofilm formation of *L. rhamnosus* more than PS” was accepted. These findings support the general assumption that PA nanomaterials facilitate bacterial adhesion and colonization, a phenomenon previously confirmed for bacterial species such as *S aureus*, *Staphylococcus epidermidis*, and *E. coli*.^6^ In a broader context, these results further substantiate the notion that nanofibrous materials are an effective matrix for bacterial biofilm formation.^22,23^

**Figure 1:**
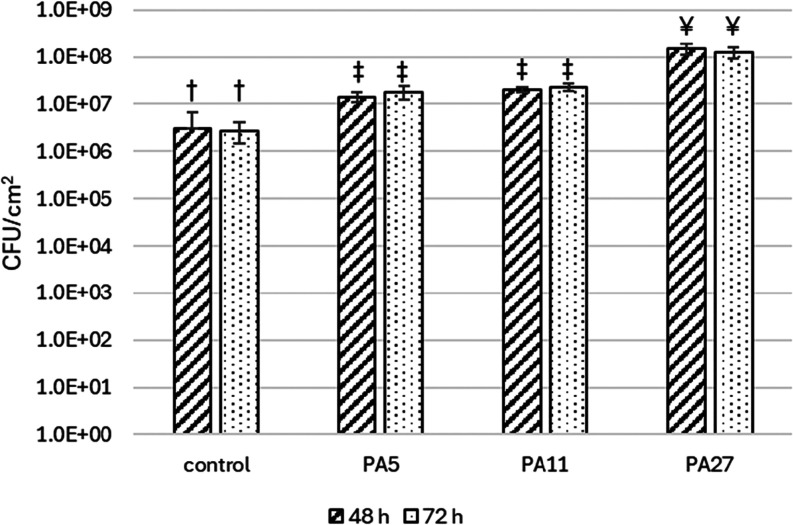
Quantitative analysis of *L. rhamnosus* biofilm on the PA nanomaterials. The CFU/cm^2^ are determined during 48 and 72 h cultivation (means that do not share a letter within the same column are statistically significant at *p* < 0.05).

The areal weight of the tested PA nanomaterials influenced the biofilm formation by *L. rhamnosus*. PA27 promoted the highest biofilm formation among the three tested materials (*p* < 0.05), achieving a biofilm CFU count of up to one logarithmic order of magnitude higher than the other two materials. This significant difference was observed for both cultivation times. No difference in biofilm formation was found between PA5 and PA11 (*p* > 0.05). The increase in the colonization for PA27 can be attributed to the higher number of spaces and compartments between the material fibers. Since PA5, PA11, and PA27 materials do not differ from each other in fiber composition or fiber diameter, the same interfibrous compartment size can be assumed for all of them, and the effect of its size on the variation in the measurement of cell growth can therefore be neglected. Although the internal structure (i.e., the arrangement of fibers) remains the same, materials with higher areal weight are thicker and therefore contain more interfibrous compartments. These compartments may serve as niches for planktonic cells to colonize, implicitly leading to greater colonization depth within the material. SEM analysis confirmed that areal weight influenced the structure and localization of biofilms within PA. In materials with lower areal weights, bacteria mainly colonized the surface, while on PA27, bacteria also colonized the insides of the PA matrix (Figure 2). However, findings from the SEM analysis should be interpreted with a degree of caution, as it primarily provides surface-level information and only limited insight into subsurface structures. For a more comprehensive evaluation of microbial colonization within the internal architecture of the material, advanced cross-sectional imaging techniques would be needed for a better understanding of internal colonization dynamics. Based on the overall results, the hypothesis **H2** “Areal weight of PA nanomaterials influences biofilm formation of *L. rhamnosus*” was accepted. These results further support the conclusions of several published studies that areal weight, along with fiber diameter, is a key parameter that can be manipulated to control cell adhesion and the colonization of materials.^6,22–24^

**Figure 2:**
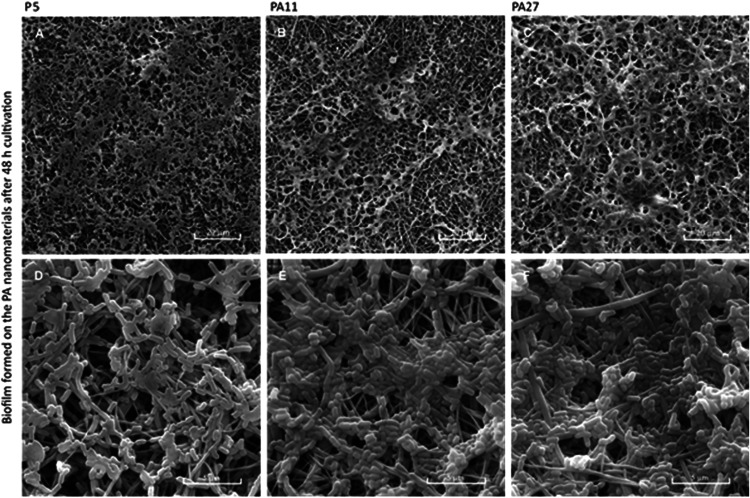
Qualitative analysis of *L. rhamnosus* biofilm on the PA nanomaterials visualized by SEM: PA5 (A, D), PA11 (B, E), and PA27 (C, E). Scale bar 20 μm (A, B, C) and 5 μm (D, E, F).

### 3.2 Tolerance Assay

The tolerance assay was designed to indicate the viability and resistance of already formed biofilms on PA, i.e., without the addition of other nutrients, enabling the multiplication of bacterial cells. To obtain a complete picture, we chose different types of tests relevant to the production, storage, and microbial safety of *L. rhamnosus* biofilm. As described above, biofilm biomass formed in 48 and 72 h did not significantly differ; thus, we performed the following tests on biofilms formed in 48 h. The hypotheses **H3** “*L. rhamnosus* cells in biofilms formed on PA remain viable longer during storage than the cells in biofilms formed on PS” and **H4** “Tolerance of *L. rhamnosus* biofilms formed on PA to external influences is impacted by areal weight” were tested.

#### 3.2.1 Biofilm Resistance under Different Storage Conditions

First, we tested the influence of storage conditions on the viability of biofilm-forming cells. The experimental storage conditions differed in terms of temperature (−20, 4, 21 °C) and environment (aqueous, dry), and the viability of biofilm-forming cells was monitored for 35 days at weekly intervals. In general, it was observed that biofilms formed on PA materials exhibited better long-term viability under all tested conditions compared to PS (*p* < 0.01), regardless of the areal weight. All tested PA materials were proven to be better scaffolds for *L. rhamnosus* biofilms than PS. Based on these findings, hypothesis **H3** “*L. rhamnosus* cells in biofilms formed on PA remain viable longer during storage than the cells in biofilms formed on PS” was accepted. This aligns with the assumption that nanofibrous materials, in general, represent more suitable materials for bacterial colonization compared to plain, nonstructured PS.^6,24,25^ As these materials mimic the extracellular matrix and, thanks to their surface/volume ratio, provide a large surface area for biofilms,^14,26^ it can be presumed that they will support strong cell adhesion and enable cells to gather into durable clusters.

However, the areal weight difference among the tested PA materials played a significant role in the biofilm cell survival (Figure 3). Biofilms formed on PA27 were the most durable under all of the tested conditions (*p* < 0.05); the biofilm (formed mainly in the structure of the material) gradually diminished during storage, and its structure changed. From day 21, the cells were already quite severely damaged (Figure 3). The highest survival rate was observed during storage in an aqueous environment at 4 °C, reaching an average of 62% of the original biofilm cell concentration before storage. Even though a significant decrease in cell numbers (Figure 3) and damage to cell shape resulting from nutrient deficiencies were observed (Figure 4), a large number of viable cells was probably assured by the colonization of the inner structures of the nanofibrous material, which provided cells with higher resistance to external influences. Additionally, dead cells serve as a nutrient source in nutrient-deficient conditions,^27^ enabling living biofilm-forming cells in thick materials to more easily reach and utilize the dead cells in their surroundings. In contrast, PA5, which had the lowest areal weight among the tested materials, showed the lowest survival rate for biofilm-forming cells. This result was expected based on previous findings that biofilm is primarily formed on the surface of this material due to its low areal weight (Figure 2). Thus, biofilms were directly exposed to stressful environmental conditions, reducing their chances of survival compared to solid associations within material structures. The higher variability is seen in specific cases, such as the survival of *L. rhamnosus* at 4 °C under dry conditions (Figure 3), likely reflecting additional sample-to-sample differences. These may include nonuniform drying rates, differences in the internal biofilm structure, or other stochastic phenomena at the cellular/population level. Because the kinetics of cell death in microbial populations are influenced by multiple random processes, including potential mutations and phenotypic shifts, some degree of variability is unavoidable.

**Figure 3:**
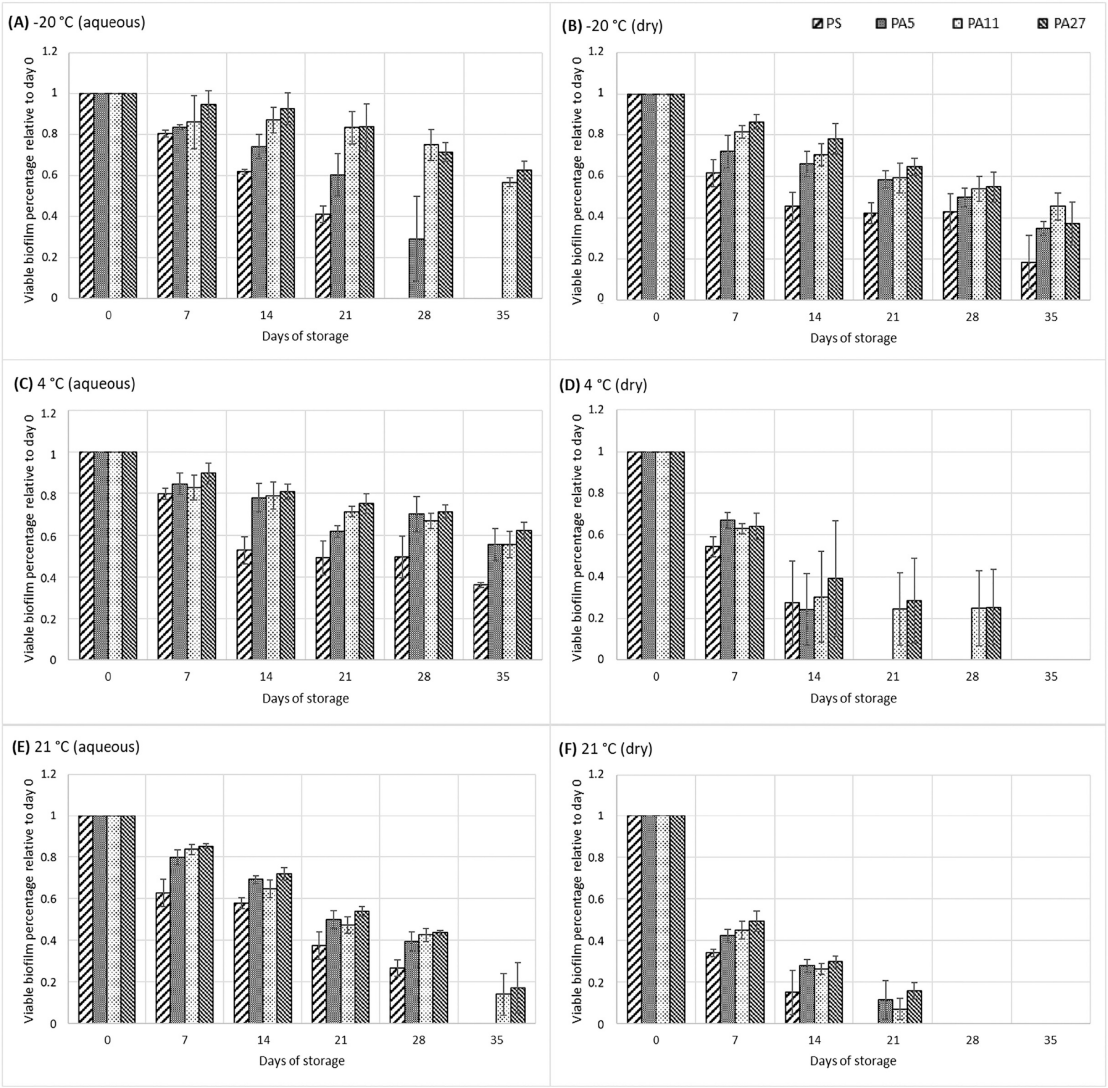
Viability of the *L. rhamnosus* biofilm cells during storage within 35 days at different temperatures (−20, 4, 21 °C) and environments (aqueous, dry). Viability is expressed as the percentage of CFU/cm^2^ related to the control. Storage conditions: (A) 20 °C, aqueous, (B) 20 °C, dry, (C) 4 °C, aqueous, (D) 4 °C, dry, (E) 21 °C, aqueous, and (F) 21 °C, dry.

**Figure 4:**
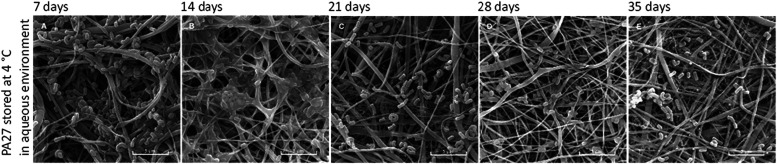
Qualitative analysis of the *L. rhamnosus* biofilm on PA27 during storage in an aqueous environment at 4 °C visualized by SEM analysis: (A) 7 days, (B) 14 days, (C) 21 days, (D) 28 days, and (E) 35 days. Scale bar: 5 μm.

Further, in the presented SEM (Figures 2, 4, and 5), differences in the amount of biofilm extracellular matrix can be noted. Extracellular matrix provides a stable and complex microenvironment, which is crucial for biofilm protection and vitality.^28^ As the production of extracellular matrix in biofilms varies among the tested PA materials, it suggests that PA’s characteristics may influence its amount and structure and consequently the overall biofilm resistance and viability. As no standardized protocol for biofilm matrix quantification on nanofibrous materials is available, it would be useful to design one and address this issue in more detail in follow-up studies.

**Figure 5:**
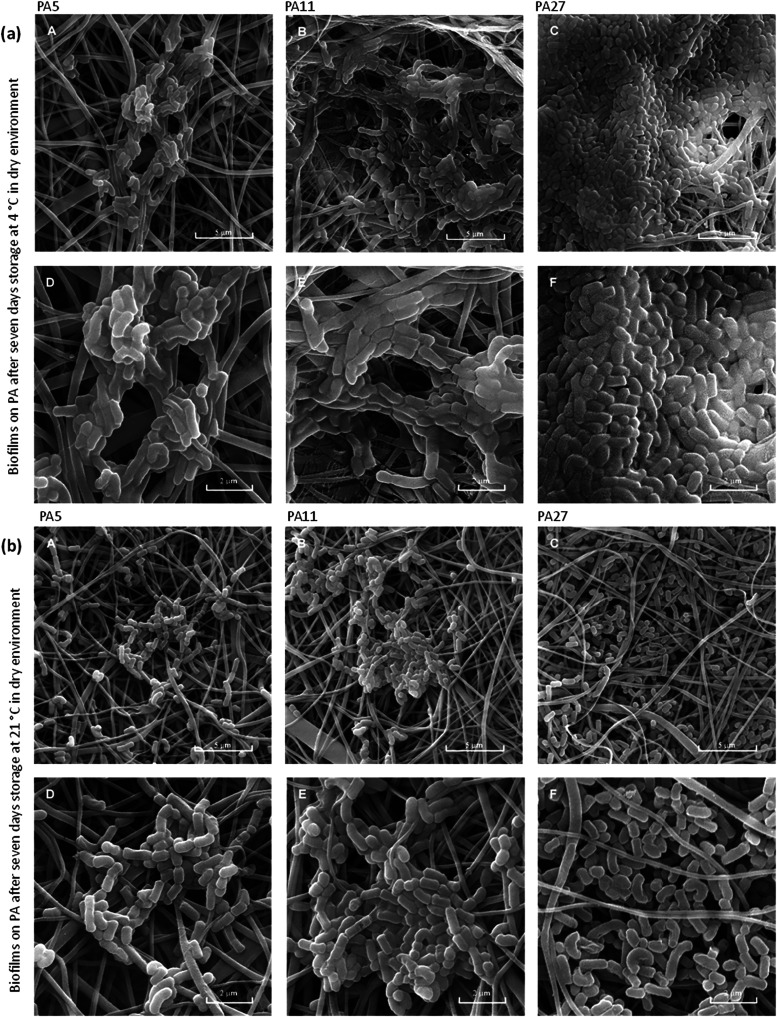
*L. rhamnosus* biofilm on PA nanomaterials stored in a dry environment at 4 °C (a) and 21 °C (b) for 7 days: PA5 (A, D), PA11 (B, E), and PA27 (C, F). Scale bar: 5 μm (A, B, C) and 2 μm (D, E, F).

Regarding the influence of the tested storage conditions on biofilm cell viability, the combination of temperature and environment was evaluated together, and the following results were observed. Temperature of 4 °C and aqueous environment were evaluated as the most viability-supporting conditions (*p* < 0.05), followed by these combinations (ranked from best to worst): (i) −20 °C in both aqueous and dry environment, with no significant difference between the environments (*p* > 0.05), (ii) 21 °C in aqueous environment, and (iii) 21 °C and dry environment.

Regarding the effect of temperature, this can be justified by the fact that a low temperature above freezing maintains cells in a dormant but viable state with decreased metabolic activity,^29^ preventing further division or rapid drying of the cells and biofilm matrix. At −20 °C, considering that no cryoprotectant was added, storage was more challenging for biofilm-forming cells. However, a high level of viability, reaching 56–62%, was maintained after 35 days of storage for two tested PA nanomaterials, PA11 and PA27. Compared to low storage temperatures, 21 °C supported cell viability the least among the tested temperatures (*p* < 0.05), likely because cells remained metabolically active, depleting available nutrients and gradually dying after their depletion. Differences in the biofilm structure after 7 days of storage at 4 and 21 °C are presented in Figure 5. It can be seen that at room temperature, the biofilm diminishes rapidly, whereas in a refrigerator, it does not lose its structure so much; PA27 always has a thicker biofilm after 7 days than the other materials, especially the thinnest one, PA5. However, the influence of the environment also plays a crucial role. In an aqueous environment, i.e., storage in saline solution, cell survival was significantly better than that in dry storage (*p* < 0.05). The smallest effect of the dry environment compared to storage in saline was observed at −20 °C; survival rates after 35 days still ranged between 35 and 45% among the tested materials, with significantly higher survival on materials with higher areal weights (*p* < 0.05). For the other two temperatures, cell loss increased significantly after 14 days of dry storage. Saline solution provides a stable native osmotic pressure around cells, enhancing their viability. On the contrary, biofilms in a dry environment desiccate, preventing resuscitation.

These results are highly valuable for applications where storage of grown biofilms is needed. Observations show the high survival of biofilm-forming cells even without regular nutrient supplementation, which could lead to even longer survival, indicating that they are beneficial in terms of low storage requirements, which is an essential premise for their practical use.^30^ However, if longer storage or higher metabolic activity is required, we recommend considering and verifying the effects of adding nutrients or other agents such as cryoprotectants. In such cases, practical difficulties increase, as these substances must again be carefully removed before using the cells.

Until now, only one study focusing on the long-term survival of lactobacilli biofilms on nanofibrous materials has been published. Hu et al.^11^ reported that cellulose acetate nanofibrous membranes were suitable carriers for *L. paracasei* biofilms with survival up to 35 days. Our study supports the general assumption that nanofibers may prolong lactobacilli survival; nevertheless, more data is needed to understand these interactions in detail.

#### 3.2.2 Biofilm Resistance to Various pH Levels

Further, we evaluated the influence of various pH levels from strongly acidic to strongly alkaline on the viability of matured biofilms. The viability of matured biofilms exposed to extreme acidic pH 2, milder conditions with pH 4, 6, and 8, and strongly alkaline pH 10 was compared with that of biofilms exposed to neutral pH 7. As lactobacilli in general are considered very durable against extreme pH conditions,^31^ we expected a high survival rate in all of the tested conditions. In all of the cases (Figure 6), the biofilm-forming cells on PA nanomaterials were more resistant to all of the tested pH values than biofilms formed on a reference material (*p* < 0.01). That was expected for the reasons mentioned above, i.e., the plain structure of PS versus the structured matrix of PA nanomaterials. The possibility of forming biofilm clusters in the inner structure of the material allows the bacterial cells to more easily survive adverse conditions from the external environment. Additionally, this is related to the lower resistance of biofilms formed on the weakest of the materials, PA5, compared to the other two, PA11 and PA27 (*p* < 0.01). As in the previous testing of the influence of storage conditions on biofilm viability, PA27 provided the greatest cell protection and the highest number of viable cells in all pH tested. Statistical analysis confirmed that areal weight influenced biofilm survivability in all of the tested pH levels (*p* < 0.05).

**Figure 6:**
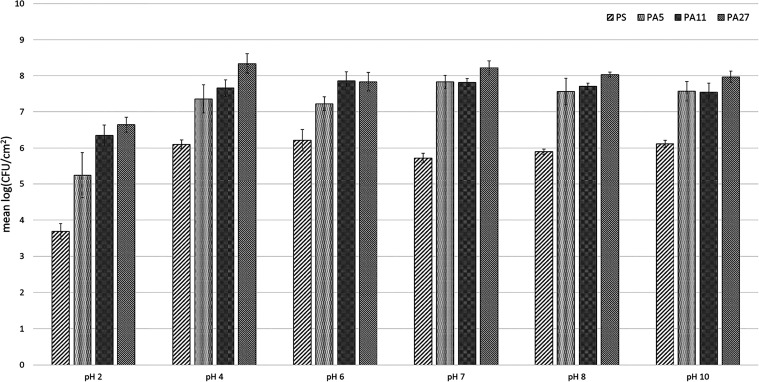
*L. rhamnosus* biofilms’ survivability on PA nanomaterials in the pH range 2–10. In all cases, biofilms on PA nanomaterials exhibited higher amounts of viable cells than the reference material, PS.

*L. rhamnosus* cells showed high tolerance to adverse pH, which is one of the presumptions for their applications as dietary supplements and permanent residence of the gut microbiota, needing to survive gastric activity and bile.^32^ At the lowest tested pH 2, up to 6.8 ± 0.03 log CFU/cm^2^ viable cells were determined; at pH 4, up to 8.2 ± 0.06 log CFU/cm^2^ survived. Such a high number of viable cells at a low pH confirms the resistance of *L. rhamnosus* to very acidic pH. Nevertheless, the difference between the effects of pH 2 and 4 is noticeable (*p* < 0.01), in favor of the higher pH level. For most organisms, pH 3 exhibits a limit under which their viability rapidly decreases.^33^ Lactobacilli’s tolerance to acidic conditions is explained by a constant gradient between extracellular and cytoplasmic pH,^32^ with F_0_F_1_-ATPase being the enzyme responsible for protecting Gram-positive microbes against acidic conditions.^33,34^ This hydrolase, which uses the energy of the proton-motive force to synthesize ATP in the inner mitochondrial membrane, is induced at a low pH and increases the intracellular pH.^30,34^ Around the neutral region (pH 6 and 8), the number of viable cells in biofilms on PA nanomaterials compared to the control biofilm at pH 7 depends on the material areal weight: for PA5, a lower number of viable cells was observed (*p* < 0.05), while for PA11 and PA27, the number was comparable to that of the control biofilm at pH 7 (*p* > 0.05). In alkaline conditions (pH 10), a still high number of viable cells was determined, with no significant difference from the neutral pH (*p* > 0.05). These results should also be interpreted in the context of biofilm physiology. Mature biofilms possess extracellular polymeric substances and internal gradients that act as buffers, reducing the immediate effect of external pH changes. Thus, the high tolerance observed in both acidic and alkaline conditions is consistent with the known protective properties of the biofilm matrix and suggests that biofilms formed on nanofiber materials with a large specific surface area could further enhance the pH resistance of the biofilm associated with these properties.

The tolerance of *L. rhamnosus* strains to extreme pH levels has been widely tested in the context of their use in dairy fermentation or mapping their viability during gastrointestinal passage. The lowest pH at which some *L. rhamnosus* strains were able to survive during short-term exposure (tens of minutes to hours) was pH 2–3, depending on the strain tested, the exposure time, and the experimental conditions.^32,35,36^ Tolerance to basic environments has been studied significantly less; the studies concluded that *L. rhamnosus* strains were able to survive several hours of exposure to a solution of pH 8.^36,37^

Compared to a large amount of research focusing on the general tolerance of lactobacilli to various pHs, studies of pH resistance of lactobacilli biofilms on nanomaterials are rare and have appeared in the literature only in the past decade. The investigation of the tolerance of *L. paracasei* biofilms formed on cellulose acetate nanofibrous membranes was published by Hu et al.^11^ The acidic and alkaline environments tested were simulated with HCl solutions at pH 1.2 and 2 and the NaOH solution at pH 10, which were left to impact the grown biofilm and planktonic cells for 10 min. The results showed that biofilm cells were significantly more resistant to acidic pH 1.2 and 2 and basic pH 10 (*p* < 0.0001) than planktonic cells, which were 3–17 times more sensitive. In another study, Hu et al.^14^ confirmed high resistance of *L. plantarum* biofilms formed on cellulose acetate nanofibrous membranes against gastrointestinal environments and proved the higher tolerance in pH 2.5–6.8 compared to planktonic cells. Our results support the general findings of these studies that nanofibrous materials may increase the resistance of lactobacilli cells to various pH levels. However, the published studies did not test different morphologies and parameters used for nanofiber production; thus, very little data are available on the possible influence of morphology on this resistance.

#### 3.2.3 Biofilm Resistance to External Bacterial Contamination

Last, we performed an analysis of *L. rhamnosus* biofilm tolerance to bacterial contamination (Figure 7). The experiments showed a clear trend of higher resistance of biofilms formed on PA nanomaterials than on PS, which was valid for both tested bacterial pathogens, *S. aureus* and *E. coli* (*p* < 0.05). Additionally, the impact of material areal weight was confirmed; biofilms formed on PA27 showed higher resistance to external contamination than biofilms formed on the other two materials (*p* < 0.05). Based on the results of these and the aforementioned analyses from the tolerance assay, the hypothesis **H4** “Tolerance of *L. rhamnosus* biofilms formed on PA to external influences is impacted by areal weight” was accepted. Furthermore, there is a difference between the ability of both the tested bacteria to disrupt the *L. rhamnosus* biofilm. However, the biofilm was relatively sparsely colonized by *S. aureus* (reaching a maximum of 3.75% of the total CFU determined in the biofilm), demonstrating a significant ability of *E. coli* to colonize the grown *L. rhamnosus* biofilm. This result can be explained by the overall higher invasiveness and resistance of Gram-negative bacteria compared to Gram-positive ones, which is determined by the differences in the cell wall structure^38^ and differences during biofilm formation, specifically different biofilm-associated amyloid-like proteins, polysaccharides, and quorum-sensing autoinducers.^39^

**Figure 7:**
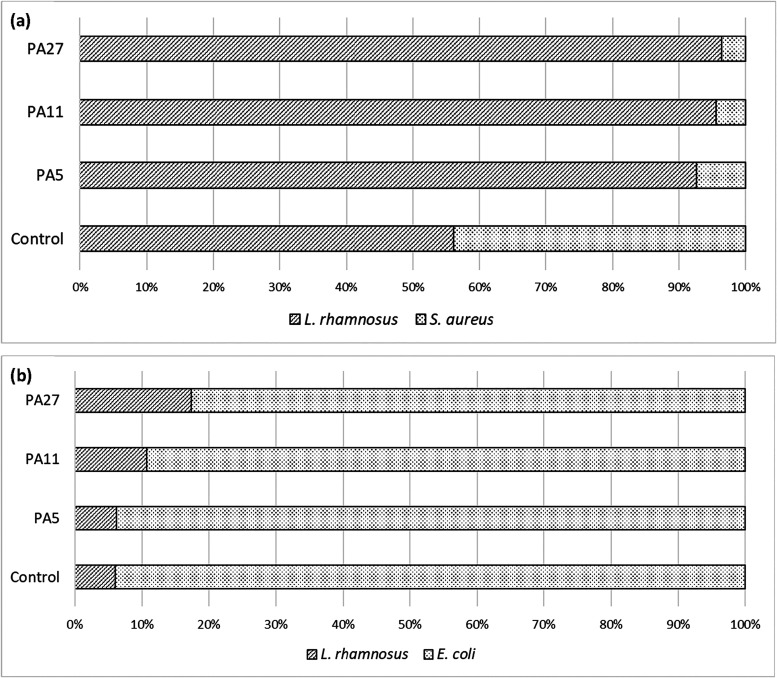
Resistance of the *L. rhamnosus* biofilm to *S. aureus* (a) and *E. coli* (b) colonization.

*L. rhamnosus* falls within lactic acid bacteria known for their strong antimicrobial effects against pathogens, such as the ESKAPE group (*E. coli*, *S. aureus*, *Klebsiella pneumoniae*, *Acinetobacter baumanii*, *Pseudomonas aeruginosa*, and *Enterococcus faecalis*),^40^ *Salmonella enterica*,^41^ *Bacillus subtilis*,^41^ *Pseudomonas putida*, and *Pseudomonas fluorescens*.^42^ The antimicrobial effect is usually attributed to the production of specific antimicrobial compounds,^43^ occupation of pathogen niches and/or displacing them,^44^ or promoting gut integrity and activation of the immune response.^40,45^ The second mechanism, the occupation of pathogen niches, is the probable reason for the strong resistance of grown biofilms to pathogen colonization studied in this paper.

To summarize the results, the tested PA nanomaterials can be considered suitable scaffolds for *L. rhamnosus* ATCC 9595 biofilm formation and storage, exhibiting a high long-term cell viability, resistance to a wide pH range, and increased resistance to bacterial contamination. The results confirm the importance of material morphology, specifically areal weight, on all of these parameters. Thus, we emphasize the need for further testing the influence of nanofiber morphology, such as fiber diameter and fiber surface structure (roughness), on cell adhesion and proliferation, as these findings can be crucial for targeted adjustments to the amount of biofilm formation as needed. This effect was already observed for eukaryotic cells, where, for example, fiber diameter and orientation influenced fibroblast morphology and proliferation on electrospun poly­(d,l-lactic-*co*-glycolic acid) meshes,^46^ and both fiber diameter and surface roughness of electrospun vascular grafts influenced blood cell activation.^47^ For prokaryotic cells, no complex data are available, despite the substantial benefits they could offer. In biotechnology, an increase in cell growth and the associated production of target substances would be highly desirable. Conversely, reducing the colonization of materials applicable (e.g., in medicine) by pathogenic bacteria appears to be essential for preventing life-threatening infections. Additionally, the different behavior of not only the polymers used to produce nanofibrous materials but also individual microbial species, as well as strains within a species, must be considered. However, despite promising potential and some progress over the past decade, there are still too few studies available to make such targeted adjustments.

## 4 Conclusions

PA nanofibrous materials are promising candidates as carriers for probiotic biofilms, which are considered the fourth generation of probiotics. In our study, three PA nanomaterials with varying areal weights were found to be more effective matrices for *L. rhamnosus* ATCC 9595 biofilm formation compared to the reference material, plain PS. Furthermore, these PA nanomaterials enhanced biofilm resistance during long-term storage and against external stressors, including strongly acidic and alkaline pH conditions, as well as contamination by pathogens such as *E. coli* and *S. aureus*. The results confirm the hypothesis that the morphology of nanomaterials plays a crucial role in the formation of the bacterial biofilm. Specifically, as the areal weight increases, both biofilm biomass and resistance to external challenges improve. Given the limited research in this area, further studies are necessary to deepen our understanding of the relationship between nanomaterial morphology and bacterial interactions. Such research could potentially lead to strategies for targeted modulation of bacterial colonization on these materials, which are highly valuable in biotechnology, medicine, or the food industry.
